# Case report: A case of posterior reversible encephalopathy in postpartum preeclampsia

**DOI:** 10.1097/MD.0000000000036023

**Published:** 2023-11-24

**Authors:** Joong-Dong Rho, Yeon-Hee Kim, Jae-Ho Shin, Tae Ki Kim

**Affiliations:** a Department of Ophthalmology, Kyung Hee University Hospital at Gangdong, Kyung Hee University School of Medicine, Seoul, Republic of Korea; b Department of Obstetrics & Gynecology, Uijeonbu St. Mary Hospital, The Catholic University of Korea College of Medicine, Gyeonggi-do, Republic of Korea.

**Keywords:** headache, hypertension, posterior reversible encephalopathy syndrome, postpartum preeclampsia, vasogenic edema

## Abstract

**Rationale:**

Posterior reversible encephalopathy syndrome is a neurological condition characterized by headache, convulsions, altered consciousness, and visual disturbance with specific radiological features, which is characterized by contrast enhancement in the occipital lobe on T2-weighted image. We report a case of sudden visual impairment of both eyes 6 days after childbirth diagnosed as postpartum preeclampsia and posterior reversible encephalopathy syndrome (PRES) through radiological examination.

**Patient concerns:**

A 31-year-old female patient with headache and visual disturbance visited the clinic.

**Diagnosis:**

Visual acuity was light perception in the right eye and hand motion in the left eye; pupillary light reflections of both eyes were normal. In the field of view test, the waveform was not observed in the defect pattern visual field power test, and the amplitude was greatly reduced in the visual field test. 1+ proteinuria was observed on urine test and magnetic resonance imaging showed contrast enhancement under both parietal and occipital cortex.

**Interventions:**

Hospitalization was done for blood pressure control and examination of related disease under suspicion of PRES caused by postpartum preeclampsia.

**Outcomes:**

Four weeks after diagnosis, vision and visual field defects recovered to normal, and the previously observed lesion on magnetic resonance imaging completely improved 3 months after the initial visit, and it was diagnosed as PRES.

**Lessons:**

PRES in postpartum preeclampsia can cause rapid vision and symptoms, visual field loss, and accurate follow-up diagnosis with relevant imaging and clinical patterns can improve vision.

## 1. Introduction

Postpartum preeclampsia is a rare condition that occurs between 2 days and 6 weeks after childbirth. It is characterized by hypertension and proteinuria, similar to epilepsy. Headaches are one of the most commonly observed neurological symptoms accompanying this condition.^[1]^

Posterior reversible encephalopathy syndrome (PRES), first identified by Hinchy in 1996, is a neurological condition characterized by headaches, convulsions, altered consciousness, and visual disturbance.^[2]^ Radiologically, PRES shows characteristic findings of contrast enhancement in the occipital region on T2-weighted fluid-attenuation inversion recovery (FLAIR) magnetic resonance imaging (MRI)^[3]^; however, the exact cause is unknown. A proposed mechanism is vascular edema in the white matter of the occipital region. In some cases, permanent vision damage may occur, but it usually improves over time without active treatment.^[4]^ However, distinguishing PRES from cerebral infarction using CT is challenging, and appropriate treatment may not be administered without an accurate diagnosis; therefore, differentiating PRES from other brain diseases has become increasingly important.

PRES has been previously reported in Korea; however, the main risk factors are diseases related to increased blood pressure, such as hypertension and preeclampsia. In particular, PRES caused by preeclampsia is the most common and occurs between 20 weeks of pregnancy and 48 hours after childbirth. However, it can occur between 2 days and 6 weeks after childbirth. Postpartum preeclampsia can be accompanied by neurological symptoms such as headache, vision disorders, hypertension, and proteinuria.^[1]^ While many cases of PRES have been reported in Korea, major preceding diseases are blood pressure-related diseases, such as hypertension and preeclampsia However, cases of postpartum preeclampsia are rare. Accordingly, we report a case of PRES in which symptoms and imaging lesions had completely improved during the 3-month follow-up period.

Patient has provided informed consent for publication of the case

## 2. Case report

**Figure 1.**
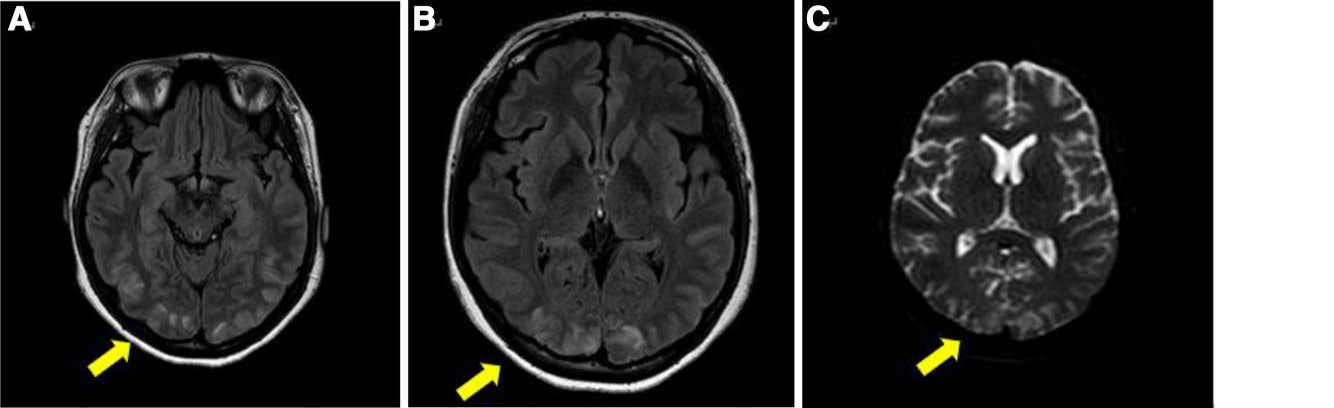
MRI performed on the day of visit. T2/FLAIR image showed high signal intensity in the parietal-temporal (A) and occipital regions (B) on both sides. High-intensity signal findings are seen on DWI image (C). DWI = diffusion-weighted imaging, MRI = magnetic resonance imaging.

A 31-year-old woman presented to our hospital with complaints of headache and visual disturbances. The headache was accompanied by a decrease in binocular vision, and its pattern seemed to be starting in both eyes and extending backward into the head. The patient was 7 days postpartum. According to her medical records, her delivery was unremarkable; however, she had gestational diabetes during pregnancy. At presentation, her blood pressure was 172/95 mm Hg, and proteinuria was 1+. Visual acuity was light perception positive (LP+) of the right eye and hand motion in the left eye; intraocular pressure measurements in the right and left eyes yielded values of 17 and 20 mm Hg, respectively. Bilateral pupil reflexes were normal. No abnormalities were observed in the anterior or posterior area of eye. Light interference tomography and computed tomography (CT) showed no specific findings; however, MRI revealed lesions with high signal strength in both the parietal and occipital regions of the brain on T2-weighted/FLAIR (Fig. 1).

**Figure 2.**
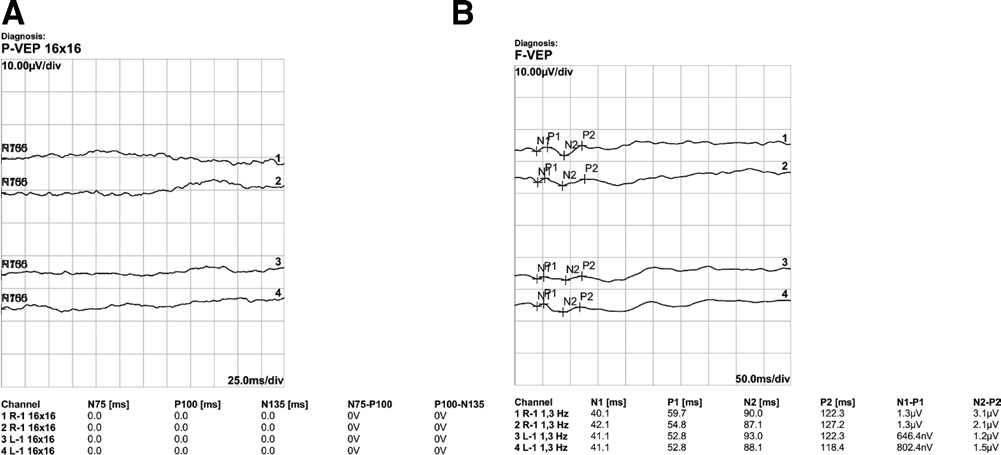
Pattern (A) and Flash (B) visual evoked potential (VEP). There was no wave in pattern VEP and the amplitude was greatly reduced in flash VEP.

The patient was hospitalized for differential diagnosis. Blood tests and ultrasound examination for blood clots were performed to differentiate cerebral infarction, revealing no abnormal findings. Fluorescent fundoscopic examination was unremarkable; however, the visual field examination revealed an exhibition deficit. No waveforms appeared during pattern visual evoked potential, and a significant amplitude reduction was found during flash visual evoke potential (Fig. 2).

**Figure 3.**
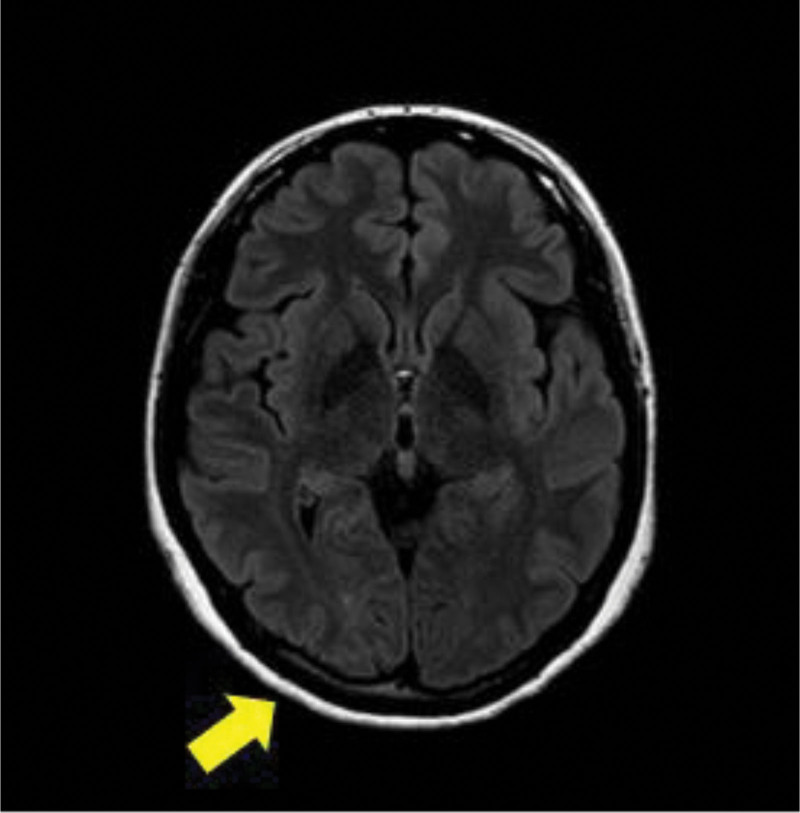
Brain MRI 3 months after onset. High signal intensity lesions in the parietal-temporal and occipital lesions disappeared in T2/FLAIR. MRI = magnetic resonance imaging.

Five days after the diagnosis, visual acuity improved to 20/32 in the right eye and 20/25 in the left eye, with no abnormalities on blood tests; the patient was diagnosed with PRES based on MRI findings, and clinical progression showed rapid recovery. Three months after onset, the patient’s headache and vision had completely improved, and the previously occurring high-signal intensity lesion disappeared on MRI conducted during 3-month follow-up visit (Fig. 3).

## 3. Discussion

PRES can be diagnosed based on symptoms of headaches, convulsions, altered consciousness, vision loss, and abnormalities such as lesions on imaging. It is rarely diagnosed in hospitals because of its varying clinical patterns. Diseases associated with PRES include epilepsy, infection, sepsis, shock, autoimmune diseases, immunocompromised disease, acute porphyria, and kidney disease.^[4]^ The most frequently reported preceding disease is epilepsy; however, accurate diagnosis and treatment are challenging because of the low frequency of postpartum preeclampsia in conjunction with absence of previous diagnosis of hypertension.^[5–7]^

The mechanism of development of PRES has not been clearly identified, but 2 hypotheses have been accepted to date.^[8]^ The first hypothesis is that the hypertension leads to abnormal cerebral vascular autonomic modulation. This causes capillary dilatation, which results in hyper-perfusion within the white matter. As the treatment for hypertension is initiated, hyper-perfusion is seen resolving during subsequent imaging examinations.^[9]^ In the second hypothesis (which was initially proposed), the hypertension causes capillaries to constrict due to automatic control of cerebrovascular vessels. This results in ischemia of the brain and vascular edema due to damage to the blood–brain barrier. This theory is supported by evidence of decreased perfusion noted on the brain MRI of patients with preeclampsia/eclampsia.^[10]^ However, both hypotheses are limited in that they cannot explain the pathophysiology of PRES without any history of hypertension.

A diagnosis of PRES is made based on the patient’s symptoms and radiological findings because diagnostic criteria have not been established. Symptoms include headaches, convulsions, altered consciousness, and decreased vision; these symptoms vary widely, making it difficult to diagnose PRES based on clinical symptoms alone.^[11]^ Computed tomography and MRI are used for the radiological diagnosis. In particular, brain MRI, which reveals a low-intensity signal from parietal and occipital lobes upon T1-weighted imaging and a high-intensity signal in T2-weighted and FLAIR imaging, is essential for the diagnosis. Diffusion-weighted imaging (DWI) and the apparent diffusion coefficient (ADC) help differentiate it from cerebral infarction because cytotoxic edema caused by cerebral infarction shows hyperintensity in DWI and hypointensity in ADC which appears as no movement of water molecules in the image; while vascular edema shows hyperintensity in DWI and ADC which appears as water retention.^[3,11–13]^

The prognosis of PRES is good but may not be so after permanent vision impairment or when presenting with other diseases.^[14]^ In general, PRES improves with symptomatic treatment, with no adverse effects, and treatment of the underlying cause. Even in our case, the patient showed progress during observation of the recovery pattern along with improvement in vision with follow-up observation alone, without specific treatment.

In this case, a patient 7 days postpartum with no relevant history during pregnancy other than gestational diabetes, experienced headache and decreased vision, hypertension, and proteinuria and was diagnosed with postpartum preeclampsia. The puerperal period involves a risk of postpartum preeclampsia and its complications. This risk should be explained to the mother, and blood pressure and neurological symptoms should be monitored. If PRES is suspected, early diagnosis is important to distinguish between vascular and cytotoxic edema by performing MRI. Early diagnosis and treatment of both diseases are important to prevent permanent neurological damage. In this case, although no history of hypertension due to headaches or rapid vision loss occurred during the puerperal period, sudden high blood pressure caused PRES; however, complete recovery was achieved through early diagnosis and blood pressure control.

## Author contributions

**Conceptualization**: Joong Dong Rho, Yeon hee Kim, Jae-Ho Shin.

**Data curation:** Joong Dong Rho, Jae-ho Shin.

**Projection administration:** Joong Dong Rho, Jae-ho Shin, Tae Gi Kim.

**Supervision:** Yeon hee Kim, Tae Gi Kim.

**Visualization:** Joong Dong Rho.

**Writing-original draft:** Joong Dong Rho.

**Writing – review & editing:** Yeon hee Kim, Jae-Ho Shin, Tae Gi Kim.
